# How does Mei-yu precipitation respond to climate change?

**DOI:** 10.1093/nsr/nwad246

**Published:** 2023-09-18

**Authors:** Bo Sun, Rufan Xue, Wanling Li, Siyu Zhou, Huixin Li, Botao Zhou, Huijun Wang

**Affiliations:** Collaborative Innovation Center on Forecast and Evaluation of Meteorological Disasters/Key Laboratory of Meteorological Disasters, Ministry of Education/Joint International Research Laboratory of Climate and Environment Change, Nanjing University of Information Science and Technology, Nanjing 210044, China; Southern Marine Science and Engineering Guangdong Laboratory (Zhuhai), Zhuhai 519080, China; Nansen Zhu International Research Centre, Institute of Atmospheric Physics, Chinese Academy of Sciences, Beijing 100029, China; Collaborative Innovation Center on Forecast and Evaluation of Meteorological Disasters/Key Laboratory of Meteorological Disasters, Ministry of Education/Joint International Research Laboratory of Climate and Environment Change, Nanjing University of Information Science and Technology, Nanjing 210044, China; Collaborative Innovation Center on Forecast and Evaluation of Meteorological Disasters/Key Laboratory of Meteorological Disasters, Ministry of Education/Joint International Research Laboratory of Climate and Environment Change, Nanjing University of Information Science and Technology, Nanjing 210044, China; Collaborative Innovation Center on Forecast and Evaluation of Meteorological Disasters/Key Laboratory of Meteorological Disasters, Ministry of Education/Joint International Research Laboratory of Climate and Environment Change, Nanjing University of Information Science and Technology, Nanjing 210044, China; Collaborative Innovation Center on Forecast and Evaluation of Meteorological Disasters/Key Laboratory of Meteorological Disasters, Ministry of Education/Joint International Research Laboratory of Climate and Environment Change, Nanjing University of Information Science and Technology, Nanjing 210044, China; Southern Marine Science and Engineering Guangdong Laboratory (Zhuhai), Zhuhai 519080, China; Nansen Zhu International Research Centre, Institute of Atmospheric Physics, Chinese Academy of Sciences, Beijing 100029, China; Collaborative Innovation Center on Forecast and Evaluation of Meteorological Disasters/Key Laboratory of Meteorological Disasters, Ministry of Education/Joint International Research Laboratory of Climate and Environment Change, Nanjing University of Information Science and Technology, Nanjing 210044, China; Collaborative Innovation Center on Forecast and Evaluation of Meteorological Disasters/Key Laboratory of Meteorological Disasters, Ministry of Education/Joint International Research Laboratory of Climate and Environment Change, Nanjing University of Information Science and Technology, Nanjing 210044, China; Southern Marine Science and Engineering Guangdong Laboratory (Zhuhai), Zhuhai 519080, China; Nansen Zhu International Research Centre, Institute of Atmospheric Physics, Chinese Academy of Sciences, Beijing 100029, China

**Keywords:** Mei-yu, extreme precipitation events, days without rainfall, water vapor, 2°C warming scenario

## Abstract

Mei-yu is an important weather phenomenon in the middle-lower Yangtze River valley (YRV) region. This study investigates the changes in the characteristics of Mei-yu under global warming and the potential reasons based on observation and reanalysis data during 1961–2022. Notable increasing long-term trends are detected in the number of days without rainfall (NDWOR), the intensity of rainfall events, and the frequency and intensity of extreme precipitation events (EPEs) in the YRV region during the Mei-yu period (15 June–10 July) over past decades. The increasing trend in NDWOR is attributed to decreased relative humidity over land surface and a longer time for the air to be replenished with moisture after rainfall events in a warming climate. The increasing trends in the intensity of rainfall events and frequency/intensity of EPEs are attributed to the strengthened transient water vapor convergence and convection in the atmosphere under global warming. Furthermore, the response of Mei-yu to 2°C of global warming with respect to the pre-industrial climate is analysed using CMIP6 models. The results suggest that the NDWOR, intensity of rainfall events and frequency of EPEs will increase in the YRV region during the Mei-yu period under the 2°C warming scenario, which implies a more challenging climate risk management in the future. Overall, the intensity of rainfall events during the Mei-yu period has the most significant response to climate change in observations and projections. The model results have a relatively large uncertainty.

## Teaser

Under global warming, the thermodynamic and dynamic effects led to increasing trends in intensity of rainfall events, frequency/intensity of extreme precipitation events, and number of days without rainfall in the Yangtze River valley during the Mei-yu period over the past six decades. Model projections suggest that these trends in Mei-yu will continue as global warming intensifies in the future with a relatively large uncertainty.

## INTRODUCTION

Mei-yu (i.e. plume rain) is a distinct weather phenomenon in East Asia during summer, which is generally characterized by persistent rainy and cloudy weather in the middle-lower Yangtze River valley (hereinafter referred to as YRV) region from mid-June to early July. Mei-yu has a broad impact on agriculture, economy and people's lives. Sufficient precipitation during the Mei-yu period is critical to the agricultural production in the YRV region during summer, autumn and winter [1,2]. In addition, increased precipitation during the Mei-yu period may increase the hydropower in the YRV region during summer and vice versa [3], where hydropower is a very important clean energy for sustainable development and for reaching the target of carbon peaking and carbon neutrality [4]. On the other hand, extreme precipitation events (EPEs) during the Mei-yu period may cause flooding and derived disasters such as debris flow, submerged cropland and damaged buildings [5–7].

Generally, the Mei-yu period in the YRV region lasts from 15 June to 10 July [8–10]. During the Mei-yu period, because of the westward extension and northward shift of the western Pacific subtropical high [10], the southerly water vapor transport marches towards the YRV region along the western flank of the western Pacific subtropical high (Supplementary Fig. S1a), which causes anomalous water vapor convergence, enhanced convection, increased clouds and persistent rainfall over the YRV region (Supplementary Fig. S1b). Climatically, the precipitation amount during the Mei-yu period accounts for 30%–40% of the annual total precipitation in the YRV region. During recent years, EPEs and persistent hot and drought events have more frequently occurred in the YRV region during the Mei-yu period, which are distinct from the typical Mei-yu weathers. For instance, in 2020, EPEs frequently occurred in the YRV region during the Mei-yu period, which caused flooding and resulted in >200 deaths/missing persons and >170 billion CNY of direct economic losses [11], whereas in 2022, persistent high temperatures and drought events occurred in the YRV region with a notably increased number of days without rainfall (NDWOR) during the Mei-yu period, which greatly affected agriculture, hydropower and human health in the YRV region [12]. These extreme events during the Mei-yu period have brought severe challenges to the government for combating climate change.

The interannual-to-interdecadal variability of the Mei-yu weathers are related to the atmospheric internal variability and ocean–land–ice–atmosphere interactions. Anomalous western Pacific subtropical high and anomalous mid-latitude westerlies over Eurasia may cause increased (decreased) water vapor convergence and intensified (weakened) convection in the troposphere, leading to increased (decreased) precipitation during the Mei-yu period [10,13–16]. These anomalous atmospheric circulations are modulated by tropical sea surface temperatures (SSTs) and sea ice/SSTs in the mid-to-high latitude oceans [11,14,17,18]. For example, the El Niño-Southern Oscillation (ENSO) and tropical Indian Ocean SST anomalies can affect Mei-yu via modulating the anomalous anticyclone over western North Pacific (WNP) [13,19,20], whereas the Arctic sea ice anomalies can affect Mei-yu via modulating the blockings over Siberia and the location of the Mei-yu front [17].

Except for the aforementioned factors of internal climate variability, the anthropogenic global warming trend since 1900 may be playing a role in affecting the long-term evolution of the weathers during the Mei-yu period [21,22]. The global atmospheric water cycle has notably changed because of global warming [23,24]. According to the Clausius–Clapeyron equation, as the atmosphere warms, its capacity to hold water increases at a rate of 7%/°C. Hypothetically, there will be more and more water vapor in the atmosphere as global warming continues [25], which may bring increased heavy rainfall if the water in the atmosphere is released. On the other hand, as the capacity of the atmosphere to hold water increases as global warming continues [26], it may become more and more difficult for the water vapor in the atmosphere to be released as precipitation, which may lead to more and more days without rainfall. However, the above hypothesis has not been examined for the weathers during the Mei-yu period. The long-term trends in Mei-yu under global warming and the reasons have not been well understood.

Furthermore, limiting global warming to 2°C above the pre-industrial levels is a long-term goal to guide all nations because the risks of extreme heat, droughts, rising sea levels, declining biodiversity and many other climate-related risks will be greatly increased when global warming exceeds 2°C [27]. Previous studies suggest that increased extreme temperature and precipitation events will occur in China under the 2°C warming scenario [28,29]. However, it still remains unclear how the Mei-yu will respond to 2°C of global warming [30], which is a crucial question that needs to be answered for better understanding the regional climate change under global warming and its potential impact on the local socio-economic development.

**Figure 1.**
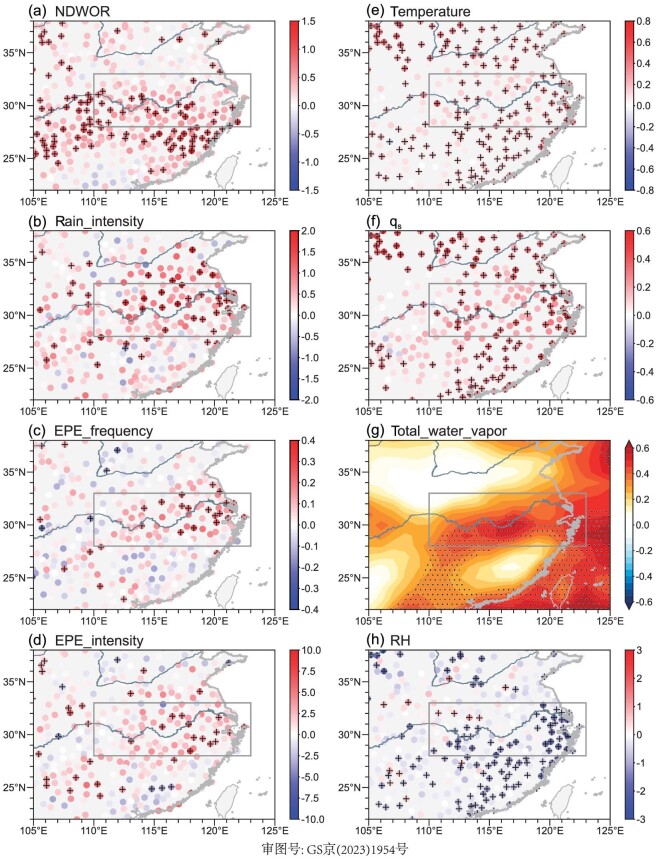
Long-term trends in factors associated with Mei-yu. Spatial distribution of long-term trends in (a) NDWOR (unit: d/10a), (b) intensity of rainfall event (unit: (mm d^−1^)/10a), (c) frequency of EPE (unit: d/10a), (d) intensity of EPE (unit: (mm d^−1^)/10a), (e) surface air temperature (unit: °C/10a), (f) surface *q_s_* (unit: (g kg^−1^)/10a), (g) total column water vapor (unit: kg/10a) and (h) RH (unit: %/10a) during the Mei-yu period during (a) 1961–2012, (b–e) and (g) 1961–2022 and (f) and (h) 1961–2020. The ‘+’ symbols in (a)–(f) and (h) and the dots in (g) denote where the trend is significant at the 90% confidence level based on the Student's *t*-test. The station data in Taiwan Province were not obtained.

Thus, in the current study, we investigate the long-term trends in the precipitation and associated factors in the YRV region (28°–33°N, 110°–123°E; Fig. 1a) during the Mei-yu period (15 June–10 July) over past decades, explore the potential reasons and further analyse the future changes of Mei-yu under the 2°C warming scenario based on the Coupled Model Intercomparison Project Phase-6 (CMIP6) models. This study aims to bring an in-depth understanding of the response of Mei-yu to global warming.

## HISTORICAL LONG-TERM TRENDS IN $\bf{Mei}\mathrm{-}\bf{yu}$

During 1961–2022, the linear trend in the time series of NDWOR is insignificant in most stations over the YRV region during the Mei-yu period (Supplementary Fig. S2a). An increasing trend during 1961–2012 and a significant interdecadal decrease during 2013–20 are observed in the time series of NDWOR (Supplementary FigsS3a and S4). Particularly, Fig. 1a shows the long-term trends in NDWOR of 105 stations in the YRV region during the Mei-yu period during 1961–2012. Most stations in the YRV region have exhibited an increasing trend in NDWOR during the Mei-yu period over the five decades before 2012. The time series of areal mean NDWOR in the YRV region during the Mei-yu period shows an increasing trend of 0.45 d/10a during 1961–2012 (Supplementary Fig. S3a). This trend is significant at the 90% confidence level based on the Student's *t*-test. A notable interdecadal decrease in NDWOR is observed in the time series of most stations in the YRV region during 2013–20 (SupplementaryFig. S2b). The ensemble empirical mode decomposition (EEMD) method [31] is used to decompose the NDWOR time series (Supplementary Fig. S3a) into different components representing interannual (Supplementary Fig. S4b), interannual-to-interdecadal (Supplementary Fig. S4c), interdecadal (Supplementary Fig. S4d) and multi-decadal (Supplementary Fig. S4e) variations and long-term trend (Supplementary Fig. S4f). The results indicate that the decrease in NDWOR during 2013–20 (SupplementaryFig. S3a) is due to an interdecadal variation (Supplementary Fig. S4d) and that the NDWOR has an increasing trend during 1961–2022 (Supplementary Fig. S4f). This long-term trend explains 1.34% of the total variance of the time series of NDWOR (Supplementary Fig. S4f).

Similarly, during 1961–2022, the intensity of rainfall events exhibited an increasing trend in most stations over the YRV region during the Mei-yu period, with the most evident increasing trends observed in the lower reaches of the YRV region (Fig. 1b). The time series of the areal mean intensity of rainfall events in the YRV region during the Mei-yu period shows an increasing trend of 0.70 (mm d^−1^)/10a during 1961–2022, which is significant at the 99% confidence level based on the Student's *t*-test (Supplementary Fig. S3b). Based on the EEMD results (Supplementary Fig. S5), the increasing trend in the intensity of rainfall events accounts for 16.19% of the total variance of the time series of the areal mean intensity of rainfall in the YRV region during the Mei-yu period (Supplementary Fig. S5e). Note that the long-term trend in the frequency of rainfall events is mostly insignificant in the YRV region during the Mei-yu period (not shown). Correspondingly, an increasing trend of total precipitation amounts have been observed in most stations of the YRV region during the Mei-yu period over the past six decades (Supplementary Fig. S6a), with a trend of 8.0 mm/10a in the time series of the areal mean total precipitation amount over the YRV region (Supplementary Fig. S6b).

Furthermore, Fig. 1c and d shows the long-term trends in the frequency and intensity of EPEs over the YRV region during the Mei-yu period over the past six decades, respectively. It can be seen that most stations in the YRV region show an increasing trend in the frequency and intensity of EPEs during the Mei-yu period, with most significant increasing trends observed in the lower reaches of YRV. The time series of the areal mean frequency (Supplementary Fig. S3c) and intensity (Supplementary Fig. S3d) of EPEs in the YRV region during the Mei-yu period had an increasing trend of 0.09 d/10a and 2.07 (mm d^−1^)/10a during 1961–2022, respectively, which is significant at the 95% confidence level based on the Student's *t*-test. The EEMD results indicate that the increasing long-term trend in the frequency and intensity of EPEs account for 10.47% (Supplementary Fig. S7f) and 11.39% (Supplementary Fig. S8f) of the total variances of the time series of the areal mean frequency and intensity of EPEs in the YRV region during the Mei-yu period, respectively.

Thus, during the past six decades, the long-term trends in Mei-yu have been characterized by increasing trends in the NDWOR, intensity of rainfall events and frequency/intensity of EPEs. These trends indicate that the weather during the Mei-yu period is becoming more unstable and extreme under global warming. Particularly, the increasing trends in the intensity of rainfall events and EPEs (NDWOR) account for a relatively large (small) portion of the variability of corresponding variables, suggesting that the increased rainfall intensity is a key feature in the response of Mei-yu to climate change. These results are basically consistent with the results of previous studies that show increasing trends of dry days and heavy precipitation events in the YRV region during summer [32–34].

## INFLUENCE OF TEMPERATURE RISING ON NDWOR

During past decades, the surface air temperature over central eastern China has exhibited a significant warming trend during summer, with a long-term trend of 0.19°C/10a during 1979–2022 [12] and with more fierce climate extremes observed against this background [34]. As shown in Fig. 1e, a notable warming trend has been observed in most stations over the YRV region during the Mei-yu period over the past six decades. The areal mean surface air temperature over the YRV region during the Mei-yu period showed a warming trend of 0.17°C/10a during 1961–2022, which is significant at the 90% confidence level based on the Student's *t*-test (Supplementary Fig. S3e). Based on the EEMD results, the increasing trend in the surface air temperature accounts for 4.26% of the total variance of the areal mean surface air temperature in the YRV region during the Mei-yu period (Supplementary Fig. S8f).

In theory, according to the Clausius–Clapeyron equation, the saturation vapor pressure (*e_s_*) and the saturation specific humidity (*q_s_*) are quasi-exponentially functions of temperature, which means that the capacity of air for holding water vapor would dramatically increase as global warming continues, at an increasing rate of ∼7% per °C rise in temperature [23]. The relative humidity (RH) of air reaching 100% is an essential condition for the occurrence of rainfall, which is given by RH = $\frac{q}{{{q}_s}}$_×_100%, where *q* is the specific humidity and *q_s_* is the saturation specific humidity.

As *q_s_* increases more dramatically than *q* under global warming [23,35], the RH is more difficult to reach 100% in many regions where there is an evident warming trend [36]. A decreasing trend in RH has been observed over continents in recent decades, which is mainly attributed to the relatively large rate of increase in *q_s_* over land due to the amplified warming over land and the relatively small rate of increase in moisture transport from ocean to land and specific humidity over land [35,37,38]. This more restrictive condition of RH for the occurrence of rainfall may lead to more days without rainfall under global warming.

During the past six decades, the surface *q_s_* has exhibited a significant increasing trend in most stations over the YRV region during the Mei-yu period (Fig. 1f), with a long-term trend of 0.21 (g kg^−1^)/10a in the time series of the areal mean *q_s_* over the YRV region during the Mei-yu period during 1961–2020 (Supplementary Fig. S3f), which is significant at the 90% confidence level based on the Student's *t*-test. Although the total column water vapor also shows an increasing trend over the YRV region during the Mei-yu period (Fig. 1g and Supplementary Fig. S3g), a decreasing trend in the surface RH is observed in most stations over the YRV region during the Mei-yu period (Fig. 1h). There was a decreasing trend of 0.47%/10a in the areal mean surface RH over the YRV region during the Mei-yu period during 1961–2020, which is significant at the 90% confidence level based on the Student's *t*-test (Supplementary Fig. S3h). The long-term trends in the *q_s_*, total column water vapor and RH account for 5.29% (Supplementary Fig. S10f), 0.83% (Supplementary Fig. S11f) and 22.83% (Supplementary Fig. S12f) of the total variances of the time series of the corresponding variables. Similarly, a decreasing trend in the RH has been observed at the 850-hPa pressure level over the YRV region during the Mei-yu period over the past six decades (Supplementary Fig. S13).

Particularly, the surface air temperature underwent an interdecadal decrease in the 2010s over the YRV region during the Mei-yu period (SupplementaryFigs S3e and S9d), which was accompanied by an interdecadal decrease in the surface *q_s_* (Supplementary FigsS3f and S10d) and an interdecadal increase in the surface RH (Supplementary FigsS3h and S12d) in the 2010s. This interdecadal variation in humidity conditions provided a relatively favorable condition for the occurrence of rainfall during the 2010s, which accounts for the interdecadal decrease in NDWOR in the YRV region during the Mei-yu period in the 2010s (SupplementaryFig. S3a). Note that the NDWOR (Supplementary Fig. S3a) and surface air temperature (Supplementary Fig. S3e) in the YRV region during the Mei-yu period rose towards a high level in 2022, whereas the time series of the *q_s_* (Supplementary Fig. S3f) and RH (Supplementary Fig. S3h) did not show variation after 2020 because the data of the *q_s_* and RH during 2021–22 were not obtained.

**Figure 2.**
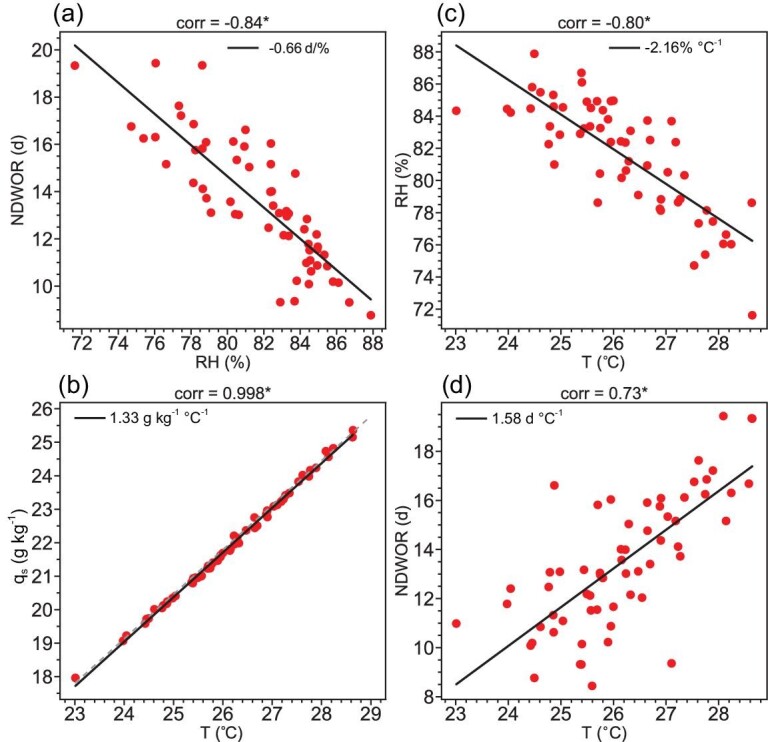
Response of NDWOR and humidity to temperature rising in the YRV region during the Mei-yu period. Scatter plot of areal mean (a) NDWOR (unit: d; *y*-axis) vs RH (unit: %; *x*-axis), (b) *q_s_* (unit: g kg^−1^; *y*-axis) vs surface air temperature (unit: °C; *x*-axis), (c) RH (unit: %; *y*-axis) vs surface air temperature (unit: °C; *x*-axis) and (d) NDWOR (unit: d; *y*-axis) vs surface air temperature (unit: °C; *x*-axis) in the YRV region during the Mei-yu period during (a) and (d) 1961–2022 and (b) and (c) 1961–2020. The black lines denote the linear regression. The gray dashed line in (b) denotes the ratio of 7%/°C according to the Clausius–Clapeyron equation.

The aforementioned increasing trend in the *q_s_* and decreasing trend in the RH means that the humidity condition has become a more restrictive condition for the occurrence of rainfall in the YRV region during the Mei-yu period under global warming. Figure 2a shows the distribution of the areal mean NDWOR versus the areal mean RH in the YRV region during the Mei-yu period over the past six decades, which indicates an evident negative correlation between the two factors, with a correlation coefficient of –0.84 significant at the 99% confidence level. The NDWOR increases by 0.66 d per 1% decrease in the RH over the YRV region during the Mei-yu period.

Furthermore, to understand the response of humidity to temperature rising in the YRV region during the Mei-yu period, Fig. 2b and c shows the distribution of the areal mean *q_s_* and RH versus the areal mean surface air temperature in the YRV region during the Mei-yu period over past six decades, respectively. The increasing rate of the *q_s_* with temperature is essentially 7%/°C according to the Clausius–Clapeyron equation (Fig. 2b). The RH decreases with temperature at a rate of 2.16%/°C (Fig. 2c). Correspondingly, the NDWOR increases with temperature at a rate of 1.58 d/°C in the YRV region during the Mei-yu period (Fig. 2d), indicating a noticeable response of the NDWOR to the temperature rising in the YRV region during the Mei-yu period.

Except for the humidity condition, the atmospheric motion is also an important factor for the occurrence of rainfall. Under global warming, the atmospheric circulation has been mostly weakened around the globe [39], especially in the mid-latitudes of the Northern Hemisphere [40]. Our results indicate that the surface wind speed in the YRV region during the Mei-yu period has been evidently decreasing over past decades (SupplementaryFig. S14a), with a decreasing trend of 0.10 (m s^−1^)/10a in the areal mean surface wind speed over the YRV region, which is significant at the 95% confidence level based on the Student's *t*-test (Supplementary Fig. S14d). This decreasing trend in the surface wind speed suggests a weakened near-surface atmospheric circulation over the YRV region during the Mei-yu period. On the other hand, an increasing trend is observed in the 850-hPa wind speed over the southern YRV region (Supplementary Fig. S14b). The different trends in the near-surface and 850-hPa wind speed may be partially because the trends in the near-surface (850-hPa) wind speed are significantly (slightly) influenced by the increased surface roughness over China due to land use and cover change [41,42]. In addition, there is a weak decreasing trend in 500-hPa vertical velocity (dp/dt), which represents intensified convection, over the lower reaches of YRV during the Mei-yu period (Supplementary Fig. S14c). Hence, the atmospheric circulation in the low- and middle-level troposphere is not weakened over the YRV region during the Mei-yu period.

Furthermore, based on observation, numerical simulations and theoretical analysis, the rainfall events, particularly the EPEs, may remove ≥7% more moisture from the air per °C of local warming and a longer time is needed for the air to be replenished with water vapor via surface evaporation and moisture advection before the next rainfall event and/or EPE occurs in a warming climate [43,44]. These processes may lead to longer dry spells and increased NDWOR [44].

Thus, the increasing trend for NDWOR in the YRV region during the Mei-yu period is mainly attributed to a more restrictive condition of RH for the occurrence of rainfall and a longer time for the air to be replenished with moisture after rainfall events in a warming climate.

## INFLUENCE OF TEMPERATURE RISING ON INTENSITY OF RAINFALL EVENTS

The global water cycle has a robust response to the global temperature rising [45,46]. The increased capacity of air for holding water vapor has led to increased precipitable water vapor over most regions around the globe [25,47], which is a favorable factor of intensified rainfall events. In the low-latitude regions, the increase in water vapor offsets the weakening of atmospheric circulation and leads to strengthened water vapor transport [24].

Particularly for weather in the YRV region during the Mei-yu period, as mentioned before, the rising temperature leads to a more restrictive condition of RH for the occurrence of rainfall. However, the amount of precipitable water increases in the atmosphere over East Asia and western Pacific under global warming (Fig. 1g), which provides a favorable condition for intensified rainfall events. In addition, the transient atmospheric circulation associated with rainfall events may be distinct from the climatic mean atmospheric circulation. Here the transient atmospheric circulations associated with rainfall events refer to the synoptic-scale atmospheric circulations concurrent with the rainfall events. For example, if a rainfall event occurred on 25 June 2020, then the transient atmospheric circulations associated with this rainfall event refer to the atmospheric circulations on 25 June 2020. The changes in transient atmospheric circulation and water vapor transport associated with the rainfall events during the Mei-yu period under global warming have not been well understood. Hypothetically, the transient atmospheric motion and associated water vapor convergence may cause a large amount of atmospheric water to be released as precipitation because of the increased water vapor content under global warming, which may result in increased intensity of rainfall events and hence increased frequency as well as intensity of EPEs.

**Figure 3.**
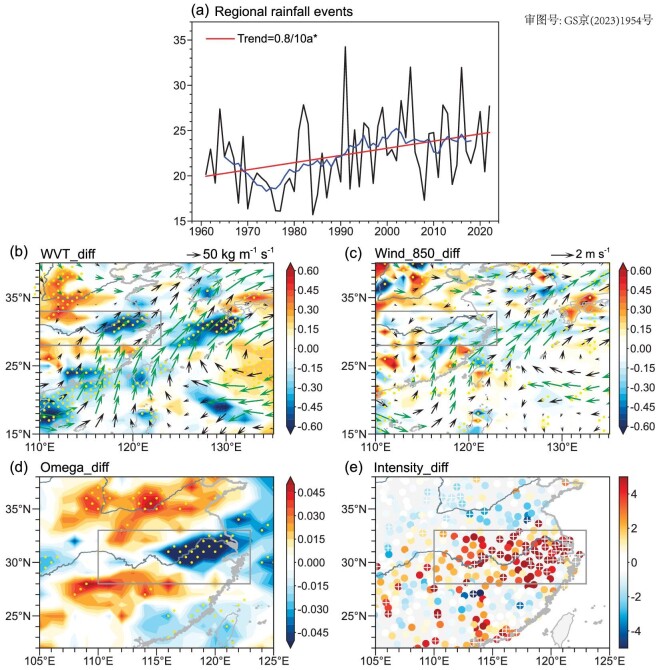
Composite differences in transient atmospheric circulations between P1 and P2 (P2 minus P1). (a) Time series of areal mean intensity of regional rainfall events in the YRV region during the Mei-yu period during 1961–2022 (units: mm d^−1^). Composite differences in transient (b) vertically integrated water vapor flux (unit: 10^−5^ kg m^−1^ s^−1^; vector) and water vapor divergence (unit: kg m^−2^ s^−1^; color), (c) 850-hPa wind (unit: m s^−1^; vector) and associated divergence (unit: 10^−6^ s^−1^; color) and (d) 500-hPa vertical velocity (unit: Pa s^−1^) associated with regional rainfall events in the YRV region during the Mei-yu period between P1 and P2 (P2 minus P1). Composite differences in the (e) intensity of regional rainfall events (unit: mm d^−1^) in the YRV region during the Mei-yu period between P1 and P2 (P2 minus P1). The red and blue lines in (a) denote the long-term trend and 9-year sliding average time series, respectively. The yellow dots in (b), (c) and (d), the vectors in green in (b) and (c), and the ‘+’ symbols in (e) denote where the differences are significant at the 90% confidence level based on the Student's *t*-test. The station data in Taiwan Province were not obtained in (e).

To examine the above hypothesis, the regional rainfall events (see Methods) characterized by a large portion of stations simultaneously having rainfall in the YRV region are identified. The time series of the intensity of regional rainfall events in the YRV region during the Mei-yu period are computed and the water vapor transport associated with the regional rainfall events during different periods are analysed. As shown in Fig. 3a, the time series of the areal mean intensity of regional rainfall events in the YRV region during the Mei-yu period exhibits an increasing trend over the past six decades. The 60 most intense regional rainfall events in the relatively cold period of 1961–80 (hereafter referred to as P1) and in the relatively warm period of 2001–22 (hereafter referred to as P2) are selected for a composite analysis. We select the first two decades (P1) and the recent two decades (P2) of the past six decades as the relatively cold period and the relatively warm period, respectively, because the surface temperatures over the YRV region and globe were evidently higher during P2 than during P1. These significant differences in the regional and global mean surface temperatures between P2 and P1 are conducive to the analysis for the changes in Mei-yu in a warming climate.

The differences in the climatic mean vertically integrated water vapor transport (Supplementary Fig. S15a), 850-hPa wind (Supplementary Fig. S15b) and 500-hPa vertical velocity (SupplementaryFig. S15c) during the Mei-yu period between the two periods are insignificant over the YRV region. However, the composite differences in the transient water vapor flux indicate that, during P2, the transient southerly water vapor transport and water vapor convergence associated with the regional rainfall events in the YRV region during the Mei-yu period were notably larger than that during P1 (Fig. 3b). This enhanced southerly water vapor transport originated from the western North Pacific and the Bay of Bengal. It should be noted that the enhanced transient southerly water vapor transport and convergence were caused not only by the increased water vapor, but also by the enhanced transient southerly winds (Fig. 3c) over the YRV region. Correspondingly, the strengthened transient convections associated with regional rainfall events occurred in the troposphere over the YRV region (Fig. 3d). These strengthened transient convections may be attributed to decreased moist static stability and increased depth of the upward motion in rainfall events under climate warming [48]. As a result, regional rainfall events with evidently larger intensities occurred in the YRV region during the Mei-yu period of P2 (Fig. 3e).

Previous studies have suggested that the thermodynamic effects associated with increased atmospheric moisture availability contribute to increased precipitation intensities in a warming climate; by contrast, the dynamic effects associated with changes in atmospheric circulation are different in different regions, which leads to region-dependent changes in the precipitation intensity [48–51]. The increased atmospheric moisture availability and enhanced convection in the troposphere associated with the regional rainfall events (Fig. 3) indicate that both the thermodynamic and dynamic effects contribute to the increased intensity of rainfall events in the YRV region during the Mei-yu period.

**Figure 4.**
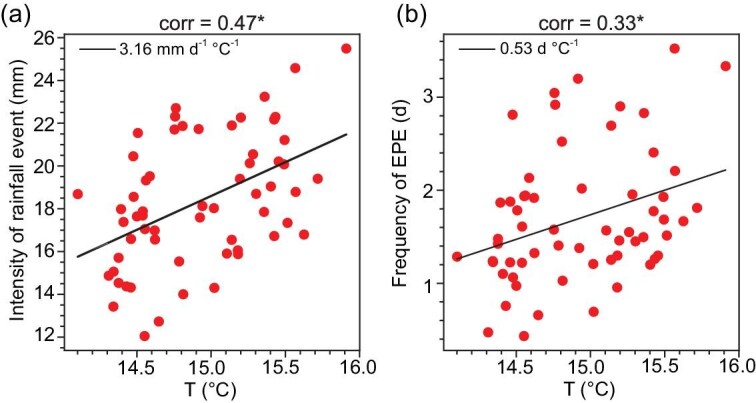
Response of intensity of rainfall events and frequency of EPEs in the YRV region during the Mei-yu period to global mean temperature rising. Scatter plot of (a) areal mean intensity of rainfall events (unit: mm d^−1^; *y*-axis) in the YRV region during the Mei-yu period vs global mean surface temperature (unit: °C; *x*-axis) during summer in 1961–2022 and (b) areal mean frequency of EPE (unit: d; *y*-axis) in the YRV region during the Mei-yu period vs global mean surface temperature (unit: °C; *x*-axis) during summer in 1961–2022. The black lines denote the linear regression.

The change in the intensity of the rainfall events and frequency of EPEs is mainly associated with the change in the large-scale atmospheric circulation and moisture under global warming. Figure 4 shows the distribution of the areal mean intensity of rainfall events and frequency of EPEs in the YRV region during the Mei-yu period versus the global mean surface temperature during summer in 1961–2022. It can be seen that the response of the intensity of the rainfall events to global temperature rising is strong, where a correlation coefficient of 0.47 is observed between the two factors, which is significant at the 99% confidence level based on the Student's *t*-test (Fig. 4a). The intensity of the rainfall events increases with the global mean surface temperature at a rate of 3.16 (mm d^−1^)/°C. Similarly, a notable response of the frequency of EPEs to the global temperature rising is also observed, where a correlation coefficient of 0.33 is observed between the two factors, which is significant at the 95% confidence level based on the Student's *t*-test (Fig. 4b). The frequency of EPEs in the YRV region during the Mei-yu period increases with the global mean surface temperature at a rate of 0.53 d/°C.

Thus, the increased intensity of rainfall events as well as the increased frequency of EPEs in the YRV region during the Mei-yu period is mainly attributed to the strengthened transient water vapor convergence and convections associated with rainfall events during the Mei-yu period under global warming.

## PROJECTION OF $\bf{Mei}\hbox{-}\bf{yu}$ UNDER 2°C WARMING SCENARIO

To understand the response of Mei-yu to 2°C of global warming, the historical and scenario (SSP1-2.6 and SSP5-8.5) experiments of 16 CMIP6 models (Supplementary Table S1) are used. The capacity of these models to reproduce the historical response of Mei-yu factors to temperature rising (Figs 2 and 4) is first examined. The results indicate that most of the CMIP6 models cannot reasonably reproduce the historical response of Mei-yu factors to temperature rising during 1961–2014 (Supplementary Fig. S16). Only 3 out of 16 models can reproduce the historical response of Mei-yu factors to temperature rising relatively well: EC-Earth3 (Supplementary Fig. S17a–c), FGOALS-g3 (Supplementary Fig. S17d–f) and ACCESS-CM2 (Supplementary Fig. S17g–i). Particularly, the simulated increases in NDWOR, intensity of rainfall events and frequency of EPEs with temperature rising are less significant in ACCESS-CM2 (Supplementary Fig. S17g–i) than that in EC-Earth3 (Supplementary Fig. S17a–c) and FGOALS-g3 (Supplementary Fig. S17d–f). However, the performance of ACCESS-CM2 is better than most of the other models in this aspect. In addition, the EC-Earth3, FGOALS-g3 and ACCESS-CM2 models can reasonably reproduce the basic features of the climatology of Mei-yu. Hence, these three models are used to analyse the future response of Mei-yu factors to 2°C of global warming.

**Figure 5.**
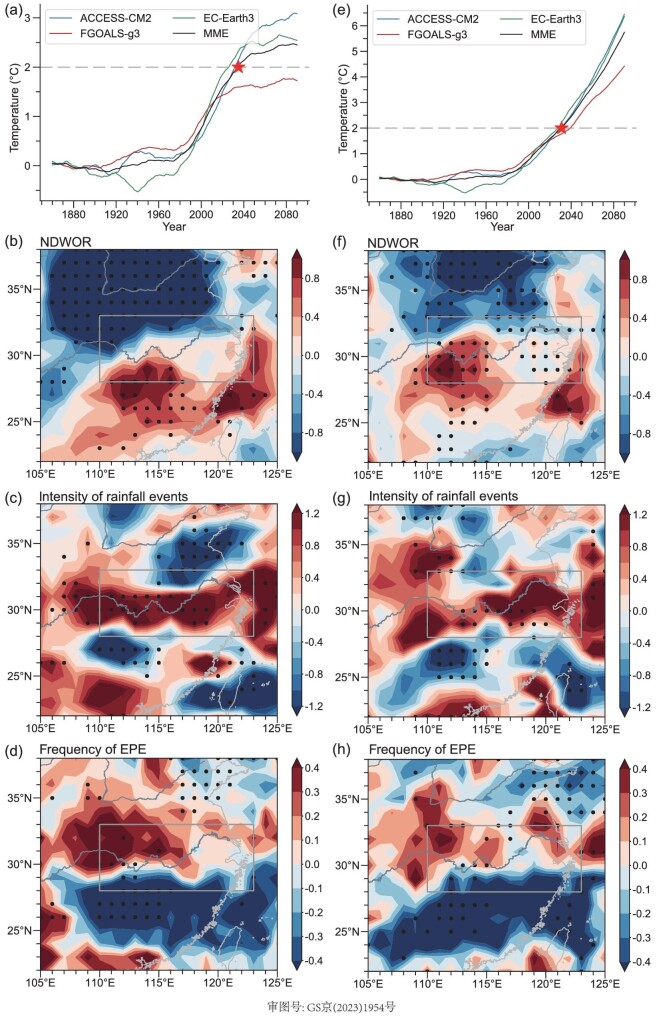
Time series of areal mean surface temperature anomalies (relative to pre-industrial levels during 1861–1900) for individual models and MME under (a) SSP1-2.6 and (e) SSP5-8.5 scenarios. Projected changes (relative to 1985–2005) of (b) and (f) NDWOR (unit: d), (c) and (g) intensity of rainfall events (unit: mm d^−1^) and (d) and (h) frequency of EPEs (unit: d) duringthe Mei-yu period under 2°C warming scenario based on (b)–(d) SSP1-2.6 and (f)–(h) SSP5-8.5 experiments. The gray dots in (b)–(d) and (f)–(h) denote where the differences are significant at the 90% confidence level based on the Student's *t*-test. The red pentacle symbols and horizontal gray dashed lines in (a) and (e) denote where the temperature rising reaches 2°C of global warming relative to the pre-industrial climate.

Figure 5a and e shows the time series of the global mean surface temperature anomalies relative to the pre-industrial climate under the SSP1-2.6 and SSP5-8.5 scenarios, respectively. The SSP1-2.6 and SSP5-8.5 scenarios represent low and high greenhouse-gas-emission pathways, respectively. The multi-model ensemble (MME) mean of the aforementioned three models indicates that the global mean surface temperature will reach 2°C of global warming in around 2035 (2031) under the SSP1-2.6 (SSP5-8.5) scenario. Correspondingly, the changes in Mei-yu during 2025–45 (2021–41) relative to the present (1985–2005) climate are examined for the SSP1-2.6 (SSP5-8.5) scenario to analyse the response of Mei-yu to 2°C of global warming.

As shown in Fig. 5b–h, the NDWOR (Fig. 5b and f), intensity of rainfall events (Fig. 5c and g) and frequency of EPEs (Fig. 5d and h) are projected to be increased in the YRV region during the Mei-yu period under the 2°C warming scenario. Specifically, the NDWOR is projected to be most significantly increased in the southwestern part of the YRV region (Fig. 5b and f). The intensity of rainfall events is projected to be most significantly increased in the central part of the YRV region (Fig. 5c and g). By contrast, the frequency of EPEs is projected to be most significantly increased in the northern part of the YRV region (Fig. 5d and h). Overall, the areal mean intensity of rainfall events over the YRV region has the largest percentage change among the three factors associated with Mei-yu, which is increased by 4.98% (4.87%) relative to the present climate under the SSP1-2.6 (SSP5-8.5) scenario. These projected changes in Mei-yu are essentially consistent with the observed changes in Mei-yu associated with global warming as mentioned previously.

Thus, as global warming intensifies in the future, Mei-yu may be further changed towards more unstable, violent and extreme weathers, which may largely increase the flood and drought disaster risk during the Mei-yu period.

## DISCUSSION

The results of this study indicate a strong response of Mei-yu in the YRV region to climate change, characterized by increased intensity of rainfall events, increased frequency/intensity of EPEs and increased NDWOR. Among these factors associated with Mei-yu, the intensity of rainfall events has the most robust response to climate change in observation and projections, whereas the response of NDWOR to climate change is less robust than that of the intensity of rainfall events and the frequency/intensity of EPEs. Thus, the robustness of the responses of NDWOR during the Mei-yu period to climate change should be more carefully examined in further studies.

The long-term trends in the factors associated with Mei-yu revealed in this study are accompanied by notable interdecadal variations. The Pacific Decadal Oscillation (PDO) can affect the interdecadal variability of Mei-yu via modulating the large-scale tropical east–west circulation during boreal summer [10]. The positive phase of the PDO generally induces enhanced convection over the YRV region during summer, which is conducive to increased rainy weather and decreased NDWOR in the YRV region during the Mei-yu period [10]. The PDO rapidly shifted from a negative phase towards a positive phase during boreal summer after 2012 (Supplementary Fig. S18), which may be an important reason for the notable interdecadal decrease in NDWOR after 2012.

The projected increases in the NDWOR, intensity of rainfall events and frequency of EPEs during the Mei-yu period under the 2°C warming scenario imply a more challenging climate risk management in the future. The relatively large uncertainty in the projected changes in Mei-yu is partially attributed to the different initial conditions and different SST biases of different models [52]. Because Mei-yu is a regional phenomenon, large ensemble high-resolution simulations are needed to reduce the uncertainty of the projection results.

## DATA AND METHODS

### Data

The daily observation data of the precipitation, surface (2-m) temperature, surface pressure, surface RH and surface wind speed of >840 stations in China are from the strictly quality-controlled meteorological element data set of China Meteorological Administration. The data of stations with missing values are removed. A total of 105 stations in the YRV region (28°–33°N, 110°–123°E) are being taken into account. The daily mean data of wind, geopotential height, vertical velocity ($\omega $) and vertically integrated water vapor flux are derived from the European Centre For Medium Range Weather Forecasts Reanalysis v5 (ERA5) [53], which have a resolution of 1°×1°. The CMIP6 data of historical and scenario experiments are derived from https://esgf-node.llnl.gov/search/cmip6/.

### Methods

The NDWOR is defined by the number of days with no precipitation during the Mei-yu period. A rainfall event is defined as the occurrence of rainfall that may last for ≥1 days with daily precipitation of >1 mm during the Mei-yu period. The intensity of rainfall events is defined as the average daily precipitation of rainfall events during the Mei-yu period. Particularly, a regional rainfall event is defined as the occurrence of a rainfall event in which >35% of the 105 stations in the YRV region simultaneously have daily precipitation of >1 mm.

The EPE is defined as the occurrence of daily precipitation exceeding the threshold of the 90th percentile of the daily precipitation during May–August in 1961–2022. The frequency of EPEs is defined as the number of days of EPEs during the Mei-yu period. The intensity of EPEs is defined as the average daily precipitation of EPEs during the Mei-yu period.

The saturation vapor pressure (*e_s_*) and the saturation specific humidity (*q_s_*) are estimated based on the air temperature using an empirical Clausius–Clapeyron equation [10,36]. The saturation vapor pressure is given by:


\begin{eqnarray*}
{e}_s = 6.11\exp\! \left[\frac{{17.56(T - 273.16)}}{{T - 35.86}}\right],
\end{eqnarray*}

where *T* is the absolute temperature. The saturation specific humidity is given by:


\begin{eqnarray*}
{q}_s = \frac{{0.622{e}_s}}{{P - 0.378{e}_s}},
\end{eqnarray*}

where *P* is the air pressure.

The linear trend of the time series of a climate variable is calculated based on a linear regression method [54]. The model is given by:


\begin{eqnarray*}
X(i) = {\beta }_0 + {\beta }_1 \times T(i) + S(i) \times {X}_{noise}(i),
\end{eqnarray*}

where *X* is the climate variable, *T* is the time variable, *β*_0_ is the intercept, *β*_1_ is the slope and the last term on the right side of the equation is the noise component. The *β*_1_ is estimated using a least-squares analysis, which is considered as the long-term trend of the climate variable in this study.

The Pearson's correlation coefficient [55] is used to quantify the linear association of two variables.

The Student's *t*-test [56] is used to examine the significance of the long-term trend, correlation coefficient and composite differences of climate variables between different periods.

The EEMD method [31] is an adaptive filter for signal decomposition based on itself. It can decompose a complex time series with multi-timescale characteristics into a finite number of oscillating components at different timescales and obtain multiple intrinsic mode functions (IMFs) and a trend component. It is suitable for the decomposition of non-linear and non-stationary time series. This method obtains multiple IMFs by continuously adding appropriate normally distributed white noise to the time series.

## Supplementary Material

nwad246_Supplemental_File
