# Supplementary material for: How does Mei-yu precipitation respond to climate change?
Source: Natl Sci Rev. 2023 Sep 18;10(12):nwad246. doi: 10.1093/nsr/nwad246 (PMC10632799; doi:10.1093/nsr/nwad246)
Supplement: nwad246_Supplemental_File [file nwad246_supplemental_file.pdf]

Supplementary Figures

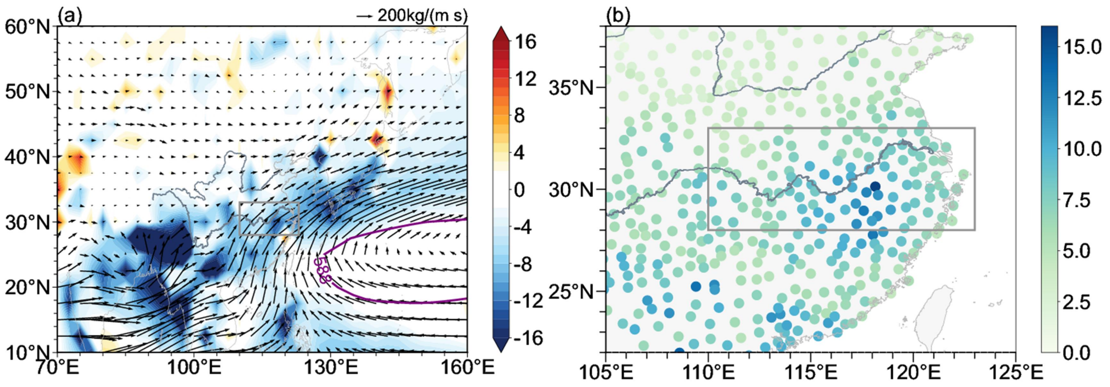

Figure S1. Climatology during the Mei-yu period. (a) Climatology of water vapor transport (unit: kg m^{-1} s^{-1}, vector) and water vapor divergence (unit: 10^{-5} kg m^{-2} s^{-1}, color), and (b) precipitation (unit: mm d^{-1}) during June 15-July 10 during 1961–2022. The purple contour in (a) denotes the isoline of 5880 gpm in gepotential height, which represents the western Pacific subtropical high. The station data in Taiwan Province is not obtained in (b).

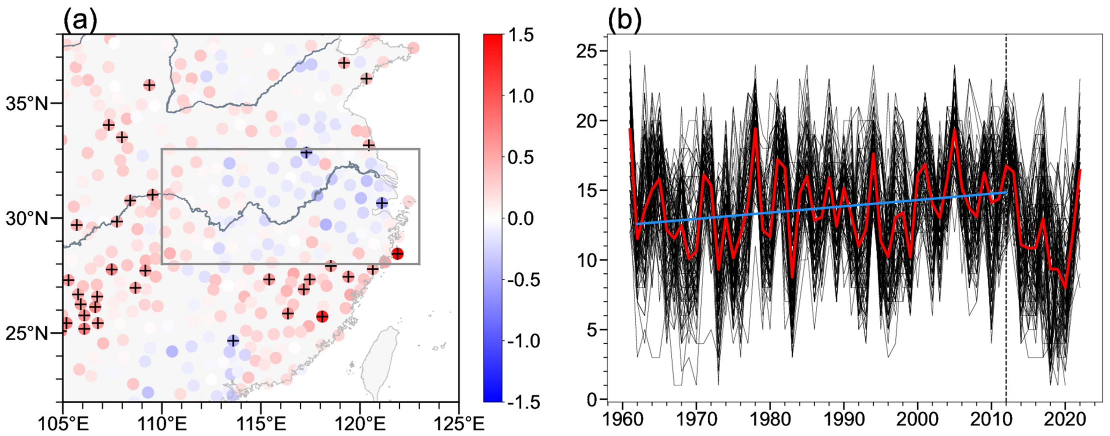

Figure S2. (a) Spatial distribution of long-term trends in NDWOR (unit: d/10a) during the Mei-yu period during1961–2022. (b) Time series of NDWOR (unit: d) in stations over the YRV region during the Mei-yu period. The "+" symbols in (a) denote where the trend is significant at the 90% confidence level based on the *Student's* t-test. The black and red lines in (b) represent time series of individual stations in the YRV region and their mean, respectively. The blue line in (b) represents the linear trend of the mean time series during 1961–2012. The station data in Taiwan Province is not obtained in (a).

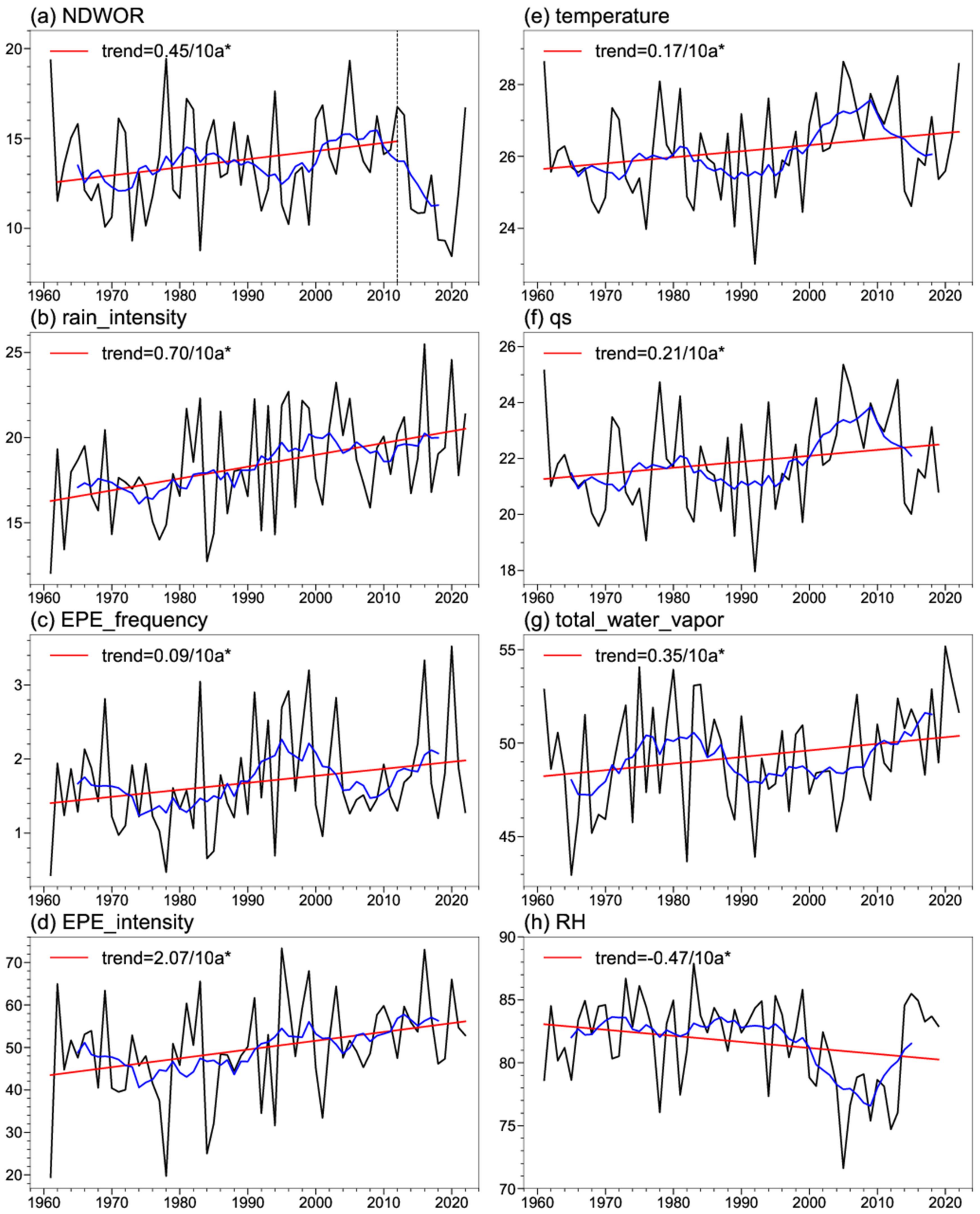

Figure S3. Time series of factors associated with Mei-yu. Time series of areal mean (a) NDWOR (unit: d), (b) intensity of rainfall event (unit: mm d^{-1}), (c) frequency of EPE (unit: d), (d) intensity of EPE (unit: mm d^{-1}), (e) surface air temperature (unit: °C), (f) surface q_s (unit: g kg^{-1}), (g) total column water vapor (unit: kg), and (h) surface RH (unit: %) in the YRV region during the Mei-yu period during (a–e, g) 1961–2022 and (f, h) 1961–2020. The red and blue lines in denote the long-term trend and the nine-year sliding average time series, respectively.

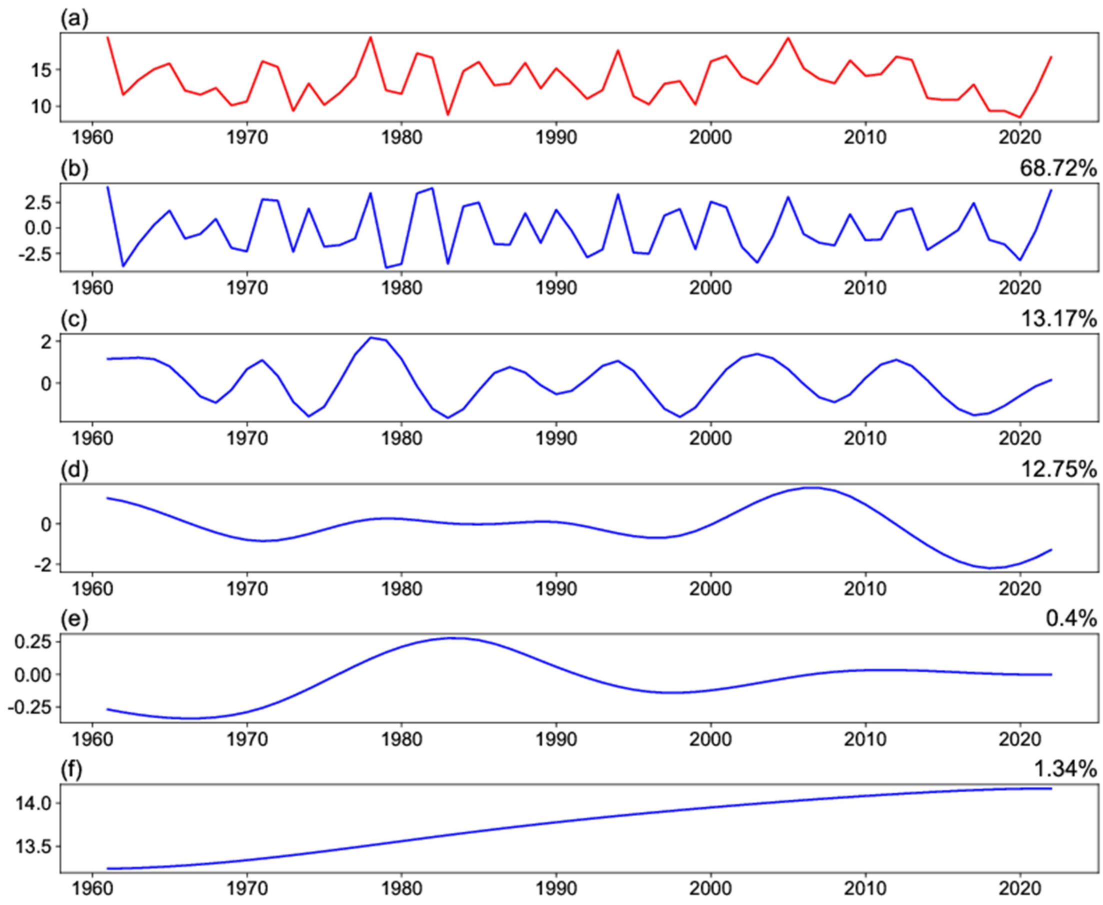

Figure S4. EEMD decomposition of time series of NDWOR. The (a) original time series, (b–e) IMFs and (f) trend components of areal mean NDWOR (unit: d) in the YRV region during the Mei-yu period based on EEMD.

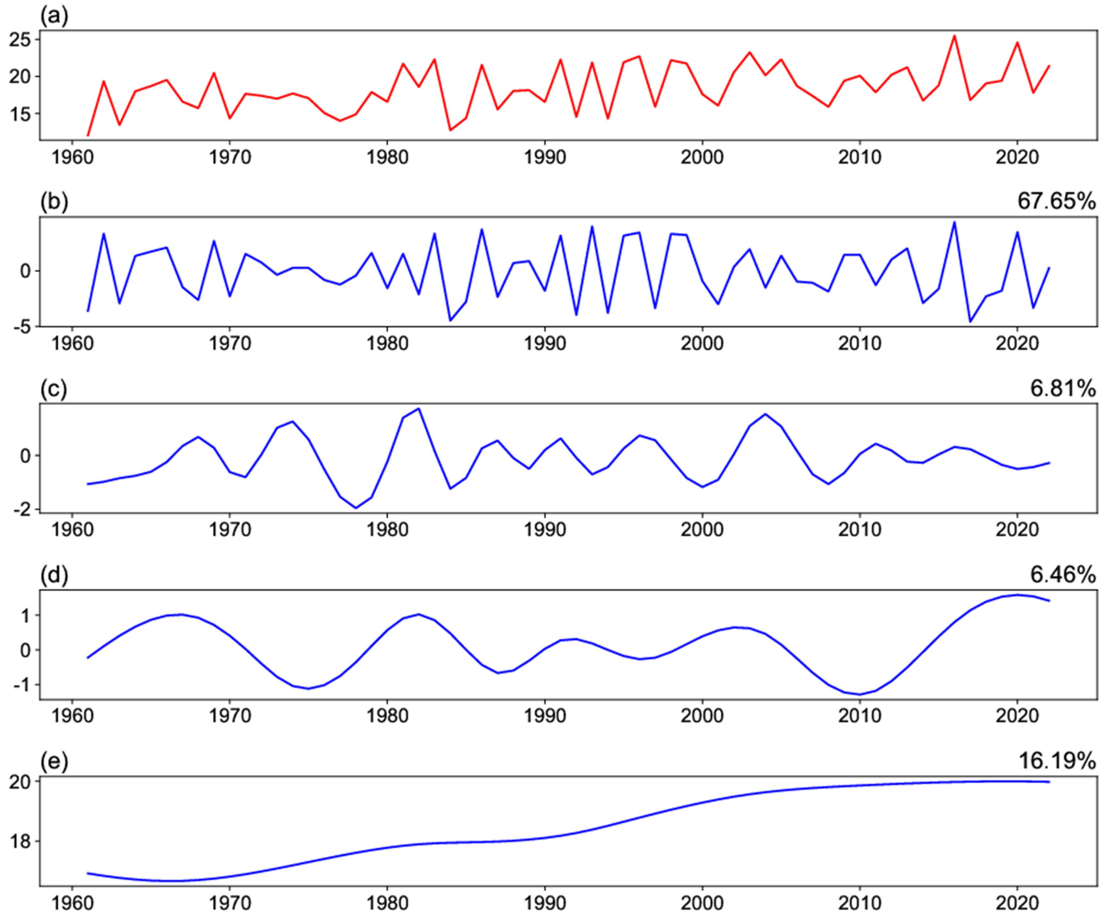

Figure S5. EEMD decomposition of time series of intensity of rainfall events. The (a) original time series, (b–e) IMFs and (f) trend components of areal mean intensity of rainfall events (unit: mm d^{-1}) in the YRV region during the Mei-yu period based on EEMD.

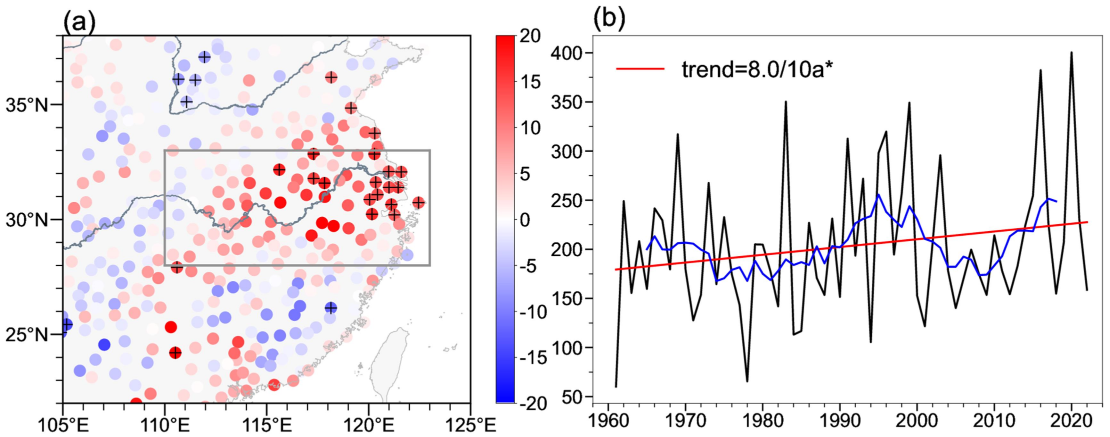

Figure S6. Long-term trend in total precipitation amount during the Mei-yu period. (a) Spatial distribution of long-term trend in total precipitation amount (unit: mm/10a) during the Mei-yu period during 1961–2022, and (b) time series of areal mean total precipitation amount (units: mm) in the YRV region during the Mei-yu period during 1961–2022. The "+" symbols in (a) denote where the trend is significant at the 90% confidence level based on the *Student's* t-test. The red and blue lines in (b) denote the long-term trend and the nine-year sliding average time series, respectively. The station data in Taiwan Province is not obtained in (a).

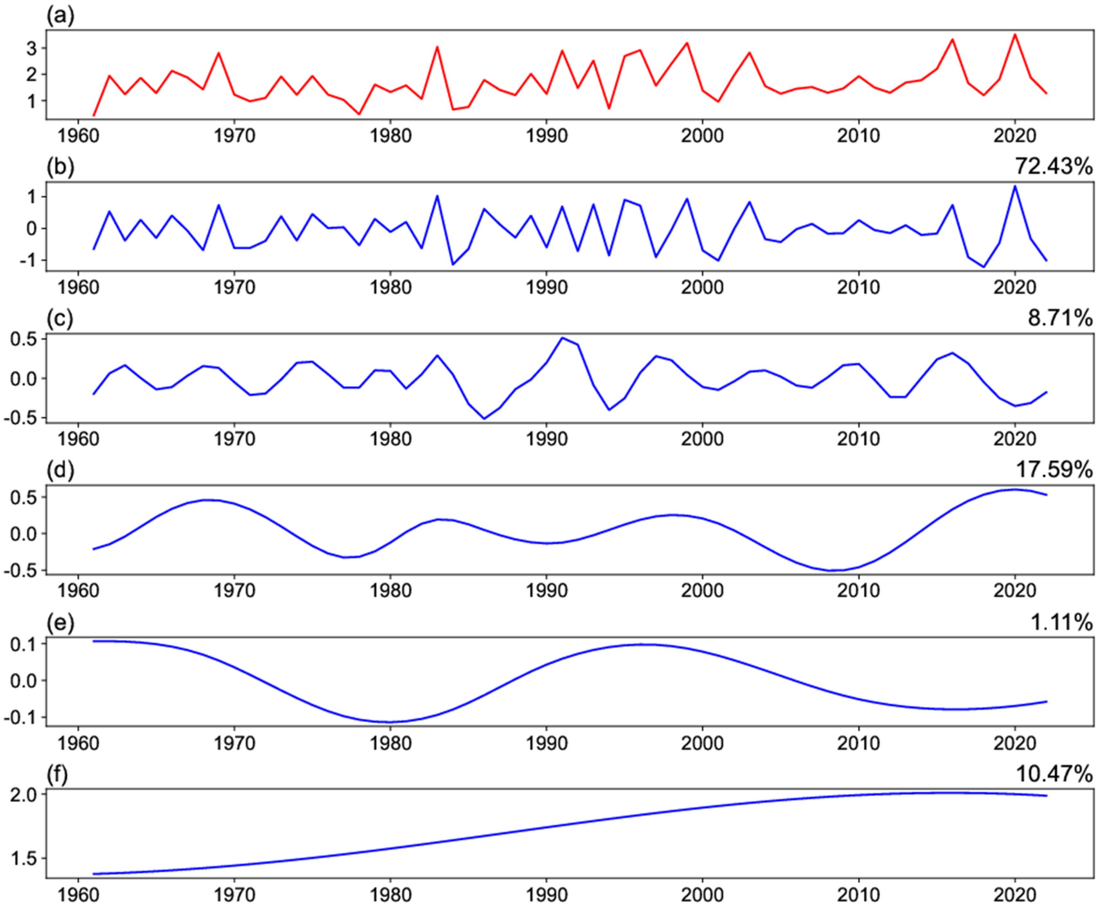

Figure S7. EEMD decomposition of time series of frequency of EPE. The (a) original time series, (b–e) IMFs and (f) trend components of areal mean frequency of EPE (unit: d) in the YRV region during the Mei-yu period based on EEMD.

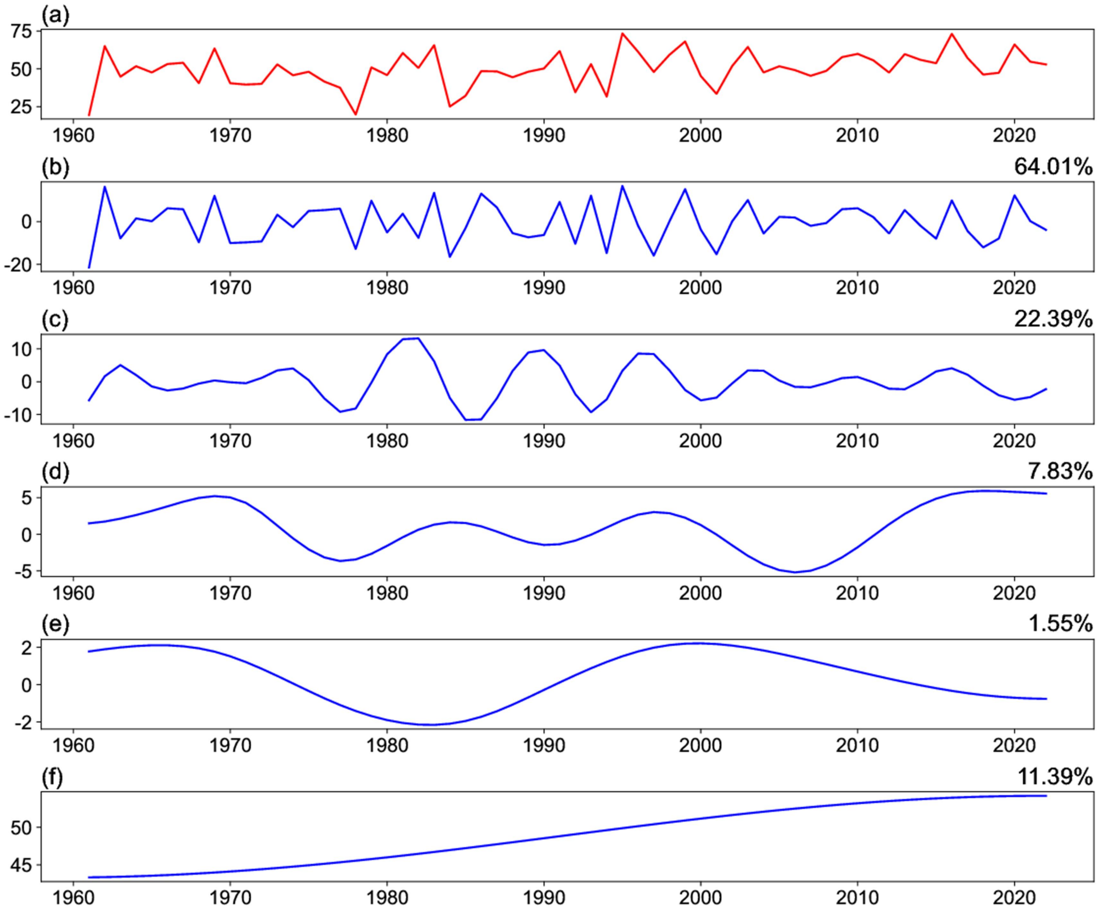

Figure S8. EEMD decomposition of time series of intensity of EPE. The (a) original time series, (b–e) IMFs and (f) trend components of areal mean intensity of EPE (unit: mm d^{-1}) in the YRV region during the Mei-yu period based on EEMD.

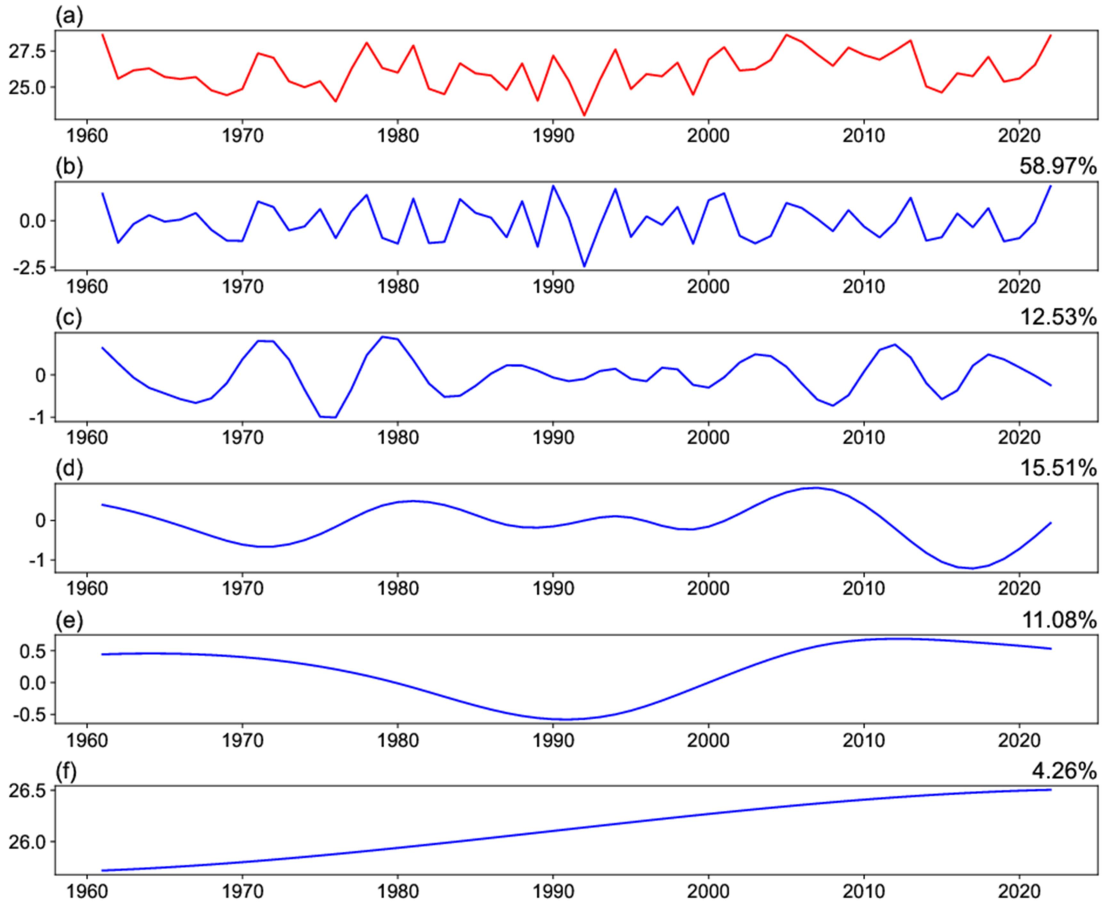

Figure S9. EEMD decomposition of time series of surface air temperature. The (a) original time series, (b–e) IMFs and (f) trend components of areal mean surface air temperature (unit: °C) in the YRV region during the Mei-yu period based on EEMD.

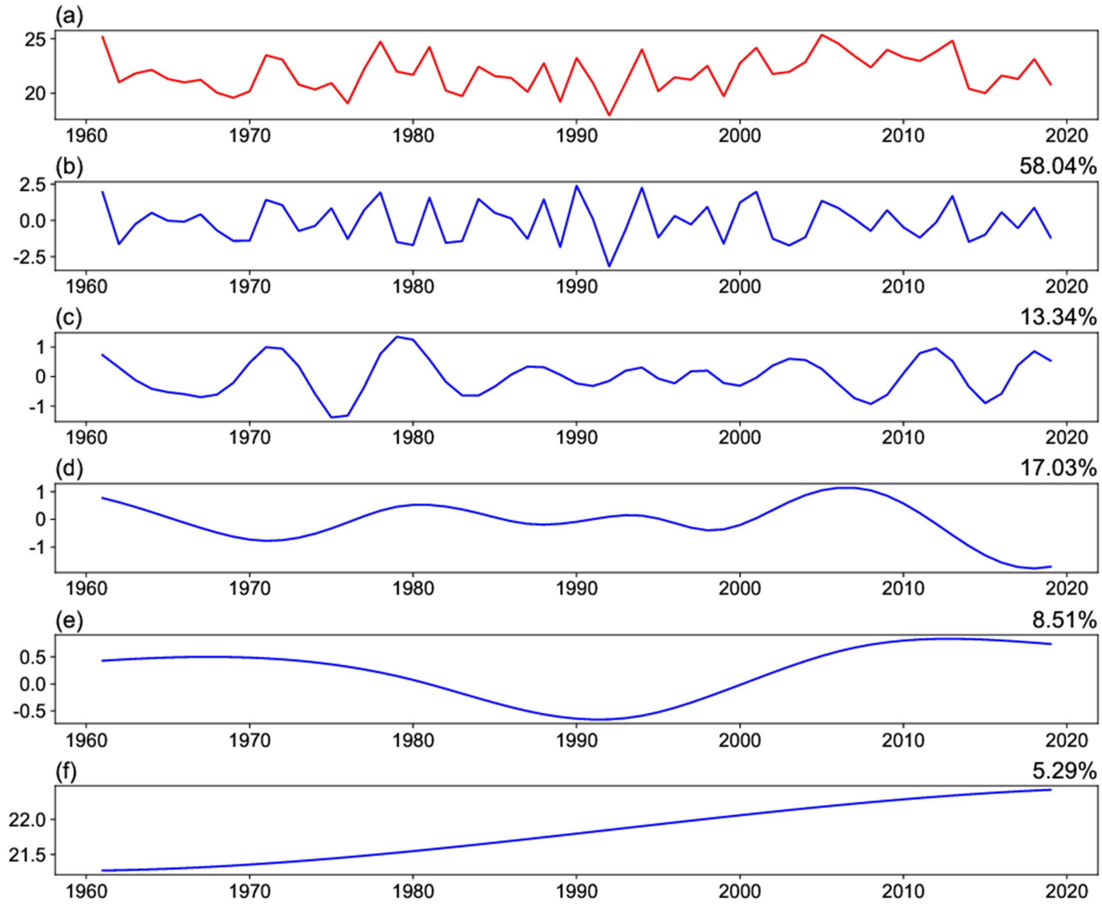

Figure S10. EEMD decomposition of time series of surface q_s. The (a) original time series, (b–e) IMFs and (f) trend components of areal mean surface q_s (unit: g kg^{-1}) in the YRV region during the Mei-yu period based on EEMD.

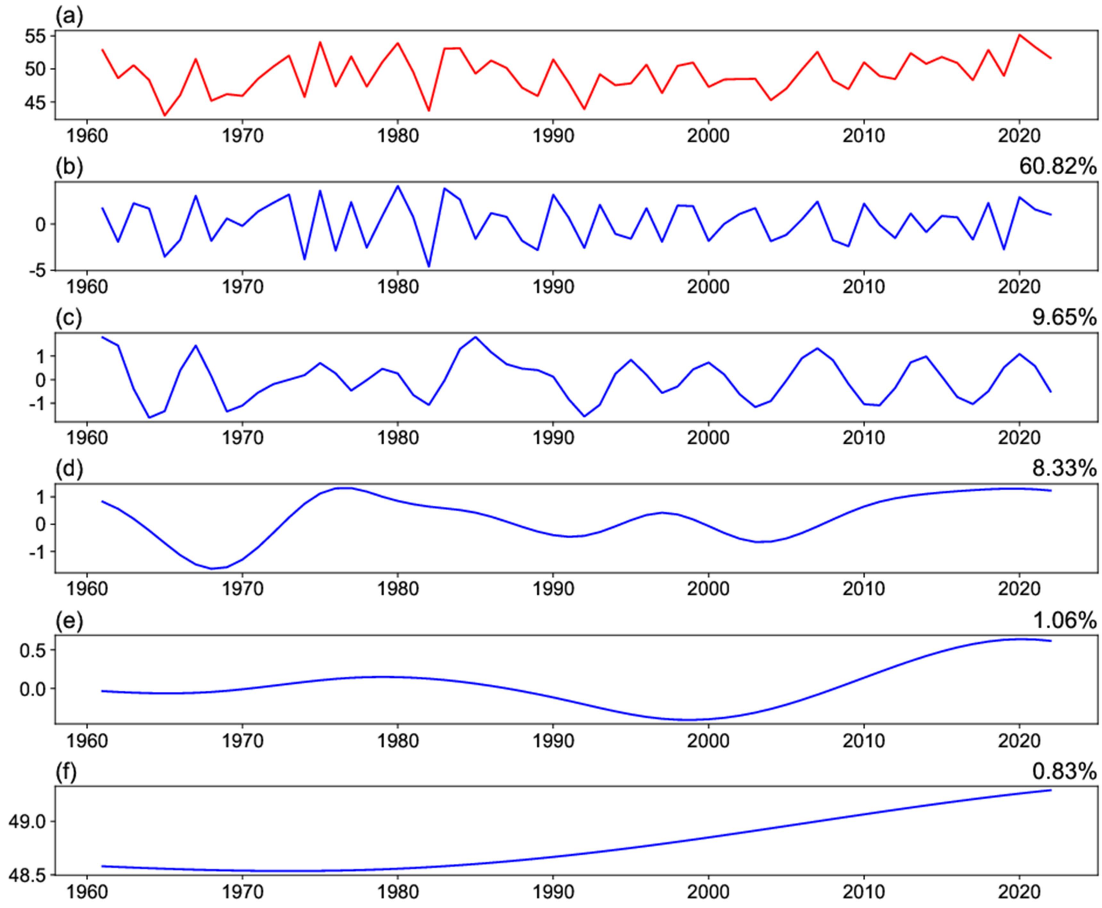

Figure S11. EEMD decomposition of time series of total column water vapor. The (a) original time series, (b–e) IMFs and (f) trend components of areal mean total column water vapor (unit: kg) over the YRV region during the Mei-yu period based on EEMD.

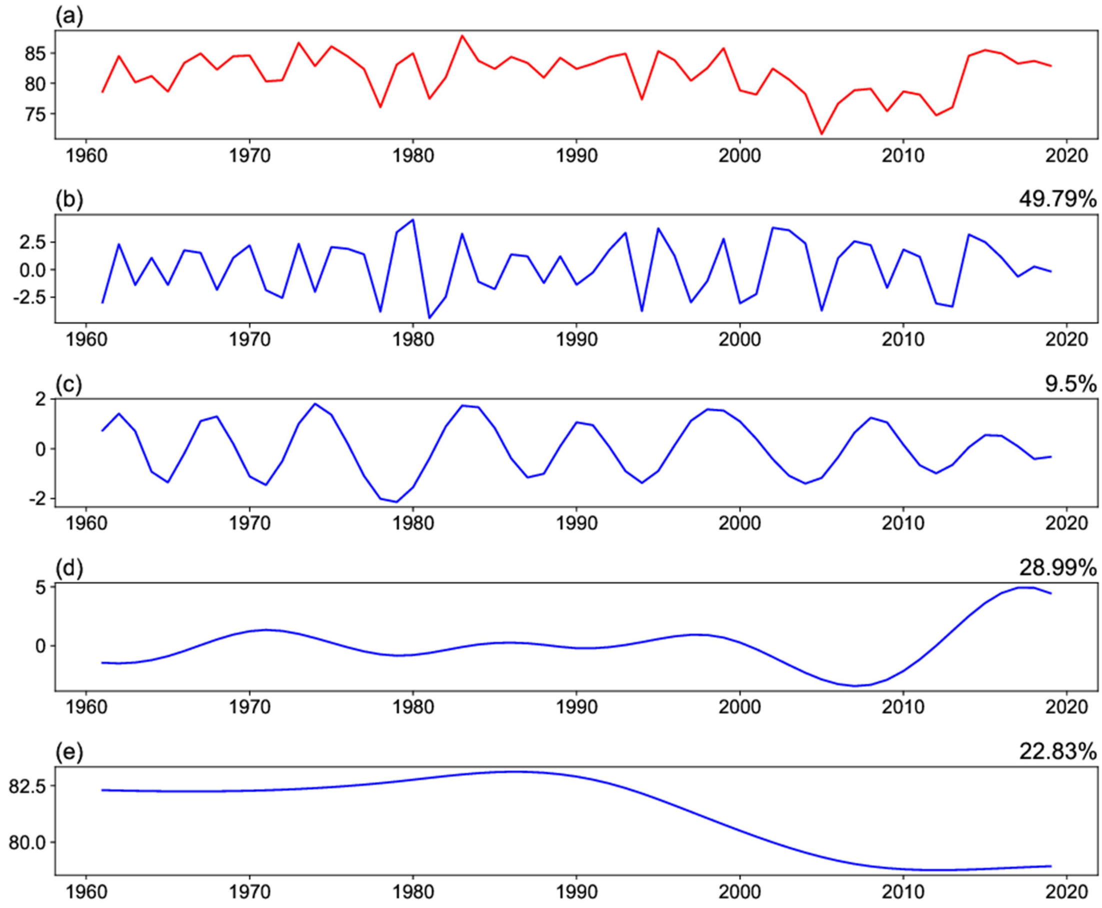

Figure S12. EEMD decomposition of time series of surface RH. The (a) original time series, (b–e) IMFs and (f) trend components of areal mean surface RH (unit: %) over the YRV region during the Mei-yu period based on EEMD.

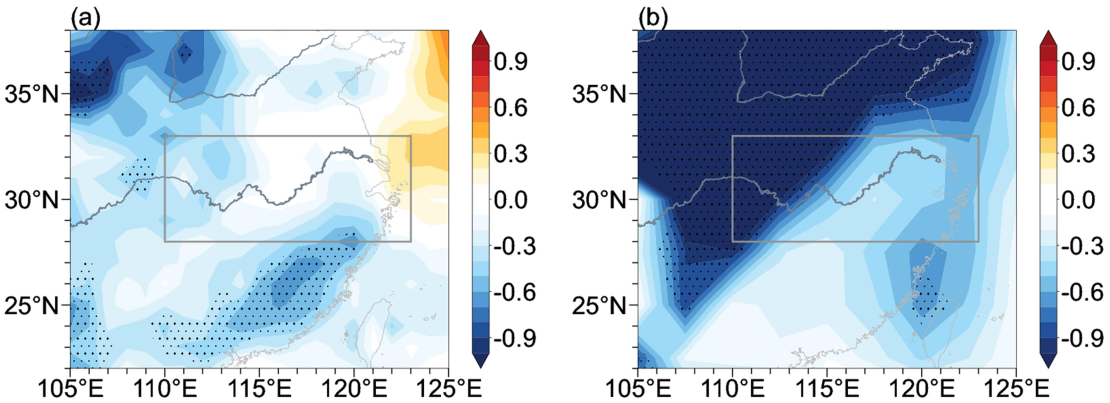

Figure S13. Long-term trends in RH (unit: %/(10a)) at the 850-hPa pressure level during the Mei-yu period during 1961–2022 based on the (a) ERA5 and (b) NCEP reanalysis data. Stippling denotes where the trend is significant at the 95% confidence level based on the *Student's* t-test.

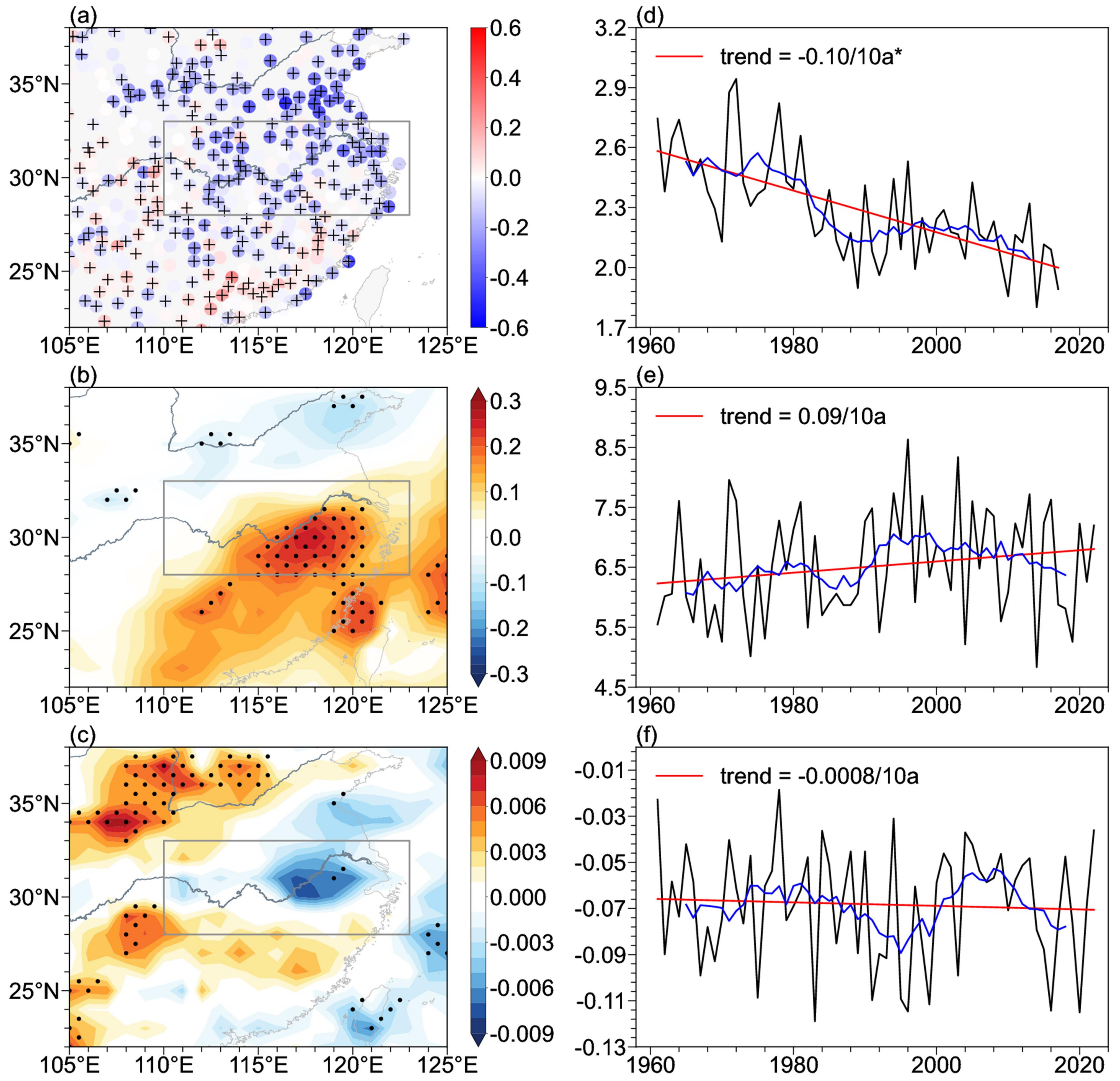

Figure S14. Long-term trends in wind speed and vertical velocity during the Mei-yu period. Spatial distribution of long-term trend in (a) surface wind speed (unit: (m s^{-1})/10a), (b) 850-hPa wind speed (unit: (m s^{-1})/10a), and (c) 500-hPa vertical velocity (unit: (Pa/s)/10a) during the Mei-yu period during (a) 1961–2017 and (b, c) 1961–2022. Time series of areal mean (d) surface wind speed (unit: m s^{-1}), (e) 850-hPa wind speed (unit: m s^{-1}), and (f) 500-hPa vertical velocity (unit: Pa s^{-1}) over the YRV region during the Mei-yu period during (d) 1961–2017 and (e, f) 1961–2022. The "+" symbols in (a), (b), and (c) denote where the trend is significant at the 90% confidence level based on the *Student's* t-test. The red and blue lines in (d), (e), and (f) denote the long-term trend and nine-year sliding average time series, respectively. The station data in Taiwan Province is not obtained in (a).

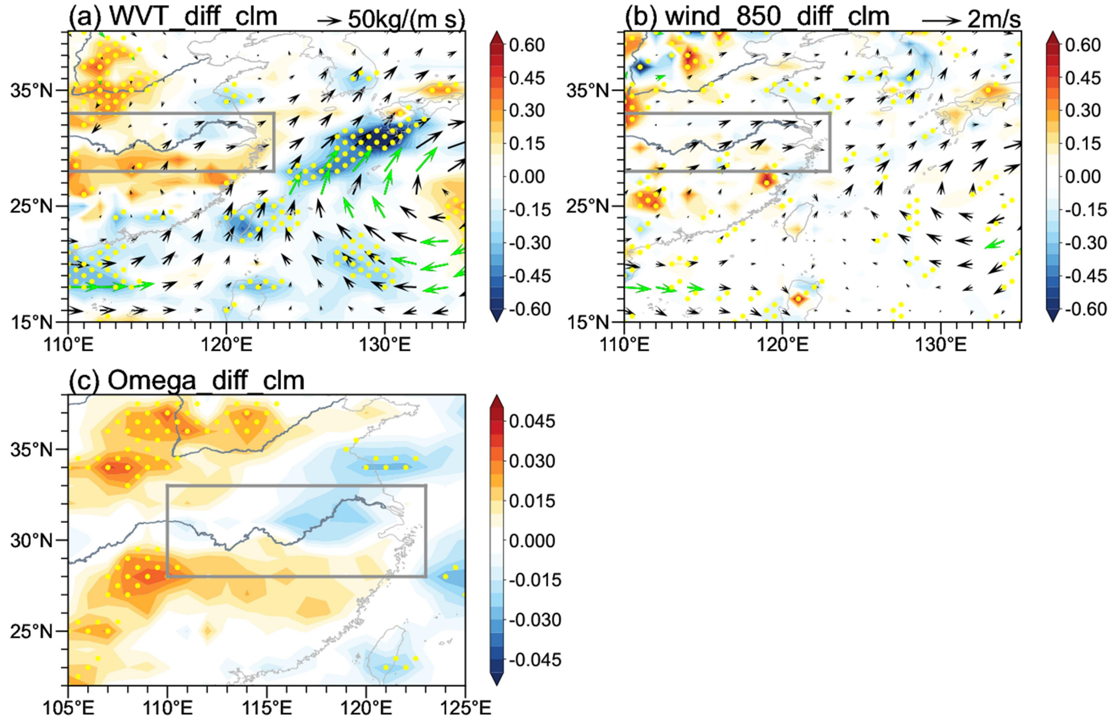

Figure S15. Composite differences in climatic atmospheric circulations between P1 and P2 (P2 minus P1). Composite differences in climatic mean (a) vertically integrated water vapor flux (unit: 10^{-5} kg m^{-1} s^{-1}; vector) and water vapor divergence (unit: kg m^{-2} s^{-1}; color), (b) 850-hPa wind (unit: m s^{-1}; vector) and associated divergence (unit: 10^{-6} s^{-1}; color), and (c) 500-hPa vertical velocity (units: Pa/s) in the YRV region during the Mei-yu period between P1 and P2 (P2 minus P1). The vectors in green and the yellow dots denote where the differences are significant at the 90% confidence level based on the *Student's* t-test.

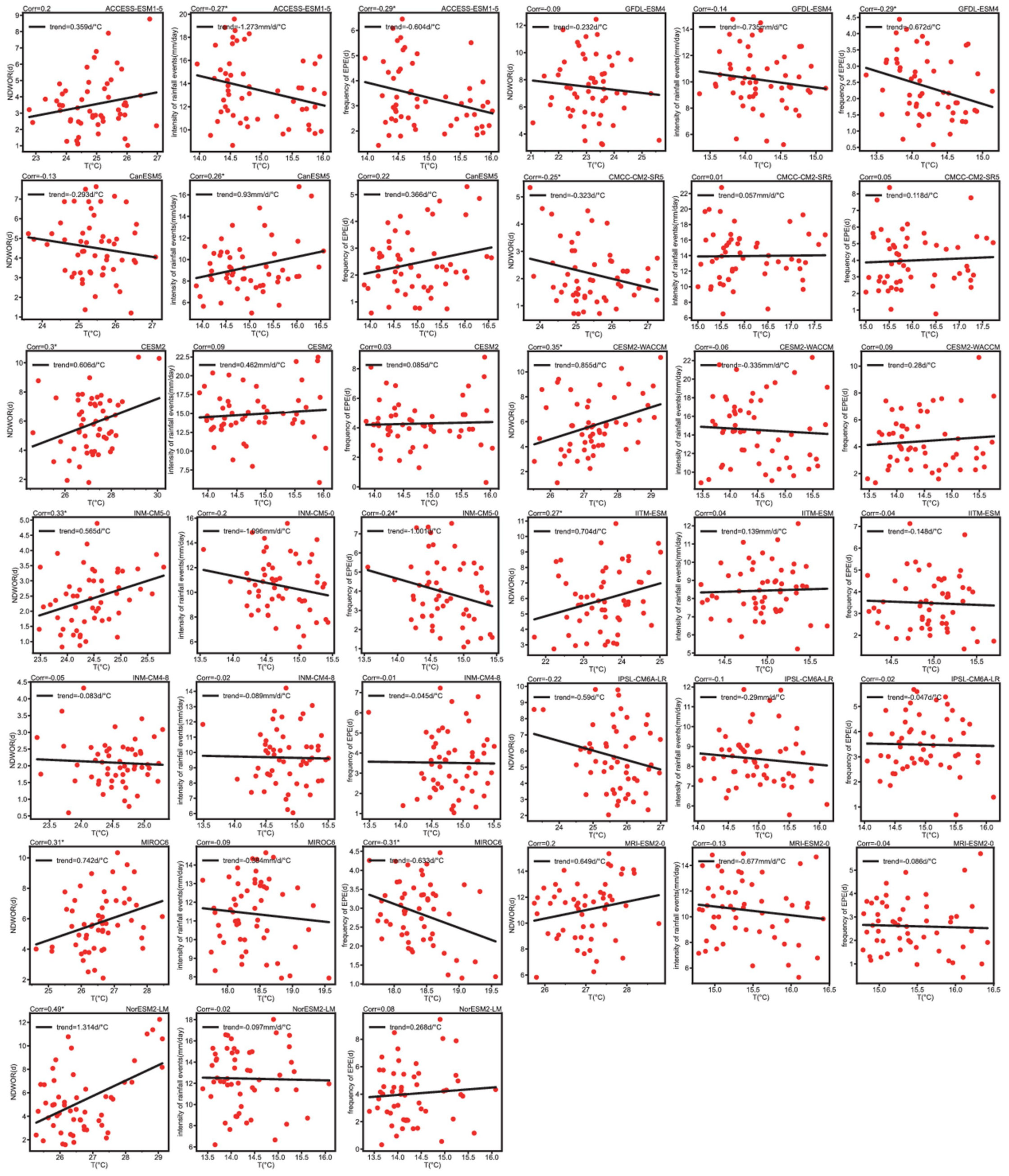

Figure S16. Simulated response of Mei-yu factors to temperature rising in CMIP6 models eliminated in this study based on historical experiments (1961–2014). The left subplot represents the scatter plot of areal mean NDWOR (unit: d; Y-axis) *vs* surface air temperature (unit: ℃; X-axis) in the YRV region during the Mei-yu period. The middle subplot represents the scatter plot of areal mean intensity of rainfall events (unit: mm d^{-1}; Y-axis) in the YRV region during the Mei-yu period *vs* global mean surface temperature (unit: ℃; X-axis) during summer. The right subplot represents the scatter plot of areal mean frequency of EPE (unit: d; Y-axis) in the YRV region during

the Mei-yu period *vs* global mean surface temperature (unit: ℃; X-axis) during summer. The black lines denote linear regression. The model names are labeled at the top-right corners of the subplots.

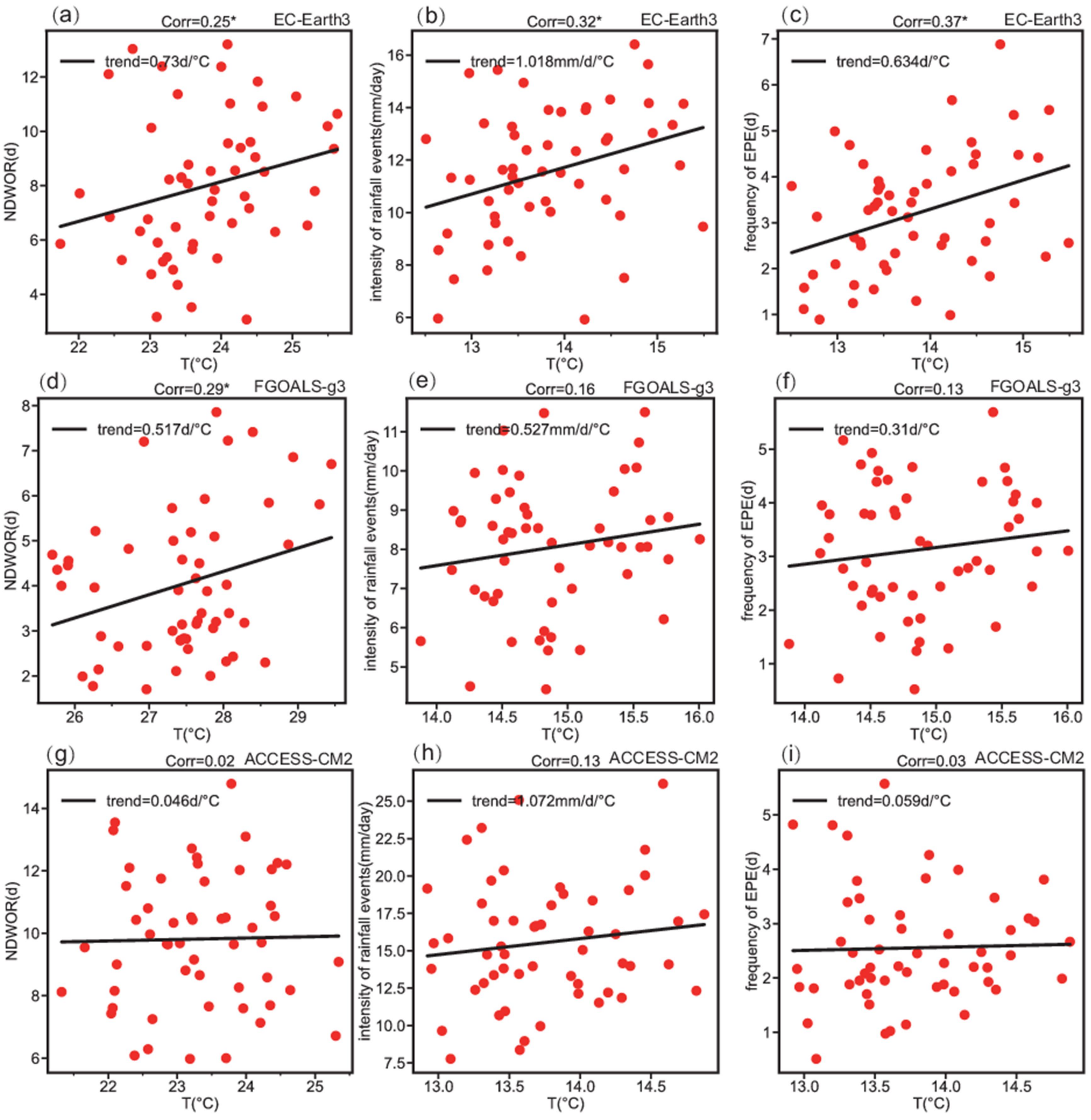

Figure S17. Simulated response of Mei-yu variables to temperature rising in CMIP6 models selected in this study based on historical experiments (1961–2014). Scatter plot of areal mean (a, d, g) NDWOR (unit: d; Y-axis) *vs* surface air temperature (unit: ℃; X-axis) in the YRV region during the Mei-yu period, (b, e, h) areal mean intensity of rainfall events (unit: mm d^{-1}; Y-axis) in the YRV region during the Mei-yu period *vs* global mean surface temperature (unit: ℃; X-axis) during summer, (c, f, i) areal mean frequency of EPE (unit: d; Y-axis) in the YRV region during the Mei-yu period *vs* global mean surface temperature (unit: ℃; X-axis) during summer. (a, b, c) EC-Earth3, (d, e, f) FGOALS-g3, and (g, h, i) ACCESS-CM2. The black lines denote linear regression.

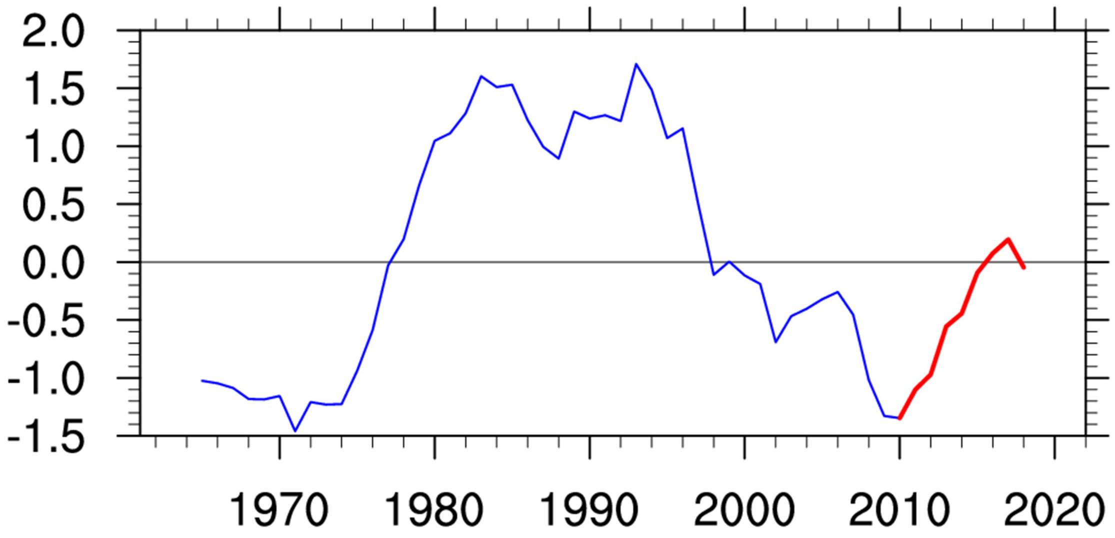

Figure S18. Standardized nine-year running mean time series of summer (May–September) Pacific Decadal Oscillation (PDO) index during 1961–2022.

Table S1. List of information for the different experiments of CMIP6 models. The models in bold indicate the preferred models.

	models	horizontal resolution	historical	SSP1-2.6	SSP5-8.5
1	**ACCESS-CM2**	192×144	r1i1p1f1	r1i1p1f1	r1i1p1f1
2	ACCESS-ESM1-5	192×145	r1i1p1f1	–	–
3	CanESM5	128×64	r1i1p1f1	–	–
4	CESM2	288×192	r1i1p1f1	–	–
5	CESM2-WACCM	288×192	r1i1p1f1	–	–
6	CMCC-CM2-SR5	288×192	r1i1p1f1	–	–
7	**EC-Earth3**	512×256	r1i1p1f1	r1i1p1f1	r1i1p1f1
8	**FGOALS-g3**	180×80	r1i1p1f1	r1i1p1f1	r1i1p1f1
9	GFDL-ESM4	288×180	r1i1p1f1	–	–
10	IITM-ESM	192×94	r1i1p1f1	–	–
11	INM-CM4-8	180×120	r1i1p1f1	–	–
12	INM-CM5-0	180×120	r1i1p1f1	–	–
13	IPSL-CM6A-LR	144×143	r1i1p1f1	–	–
14	MIROC6	256×128	r1i1p1f1	–	–
15	MRI-ESM2-0	320×160	r1i1p1f1	–	–
16	NorESM2-LM	144×96	r1i1p1f1	–	–
